# Cartilage Regeneration by Chondrogenic Induced Adult Stem Cells in Osteoarthritic Sheep Model

**DOI:** 10.1371/journal.pone.0098770

**Published:** 2014-06-09

**Authors:** Chinedu C. Ude, Shamsul B. Sulaiman, Ng Min-Hwei, Chen Hui-Cheng, Johan Ahmad, Norhamdan M. Yahaya, Aminuddin B. Saim, Ruszymah B. H. Idrus

**Affiliations:** 1 Tissue Engineering Centre, Universiti Kebangsaan Malaysia Medical Centre, Cheras, Selangor, Malaysia; 2 Department of Clinical Veterinary, Faculty of Veterinary Medicine Universiti Putra Malaysia, Serdang, Selangor, Malaysia; 3 Department of Orthopedic & Traumatology, Universiti Kebangsaan Malaysia Medical Center, Cheras, Selangor, Malaysia; 4 ENT Consultant Clinic, Ampang Putri Specialist Hospital, Ampang, Selangor, Malaysia; 5 Department of Physiology, Faculty of Medicine, Universiti Kebangsaan Malaysia, Kuala Lumpur, Selangor, Malaysia; University of Texas Southwestern Medical Center, United States of America

## Abstract

**Objectives:**

In this study, Adipose stem cells (ADSC) and bone marrow stem cells (BMSC), multipotent adult cells with the potentials for cartilage regenerations were induced to chondrogenic lineage and used for cartilage regenerations in surgically induced osteoarthritis in sheep model.

**Methods:**

Osteoarthritis was induced at the right knee of sheep by complete resection of the anterior cruciate ligament and medial meniscus following a 3-weeks exercise regimen. Stem cells from experimental sheep were culture expanded and induced to chondrogenic lineage. Test sheep received a single dose of 2×10^7^ autologous PKH26-labelled, chondrogenically induced ADSCs or BMSCs as 5 mls injection, while controls received 5 mls culture medium.

**Results:**

The proliferation rate of ADSCs 34.4±1.6 hr was significantly higher than that of the BMSCs 48.8±5.3 hr (P = 0.008). Chondrogenic induced BMSCs had significantly higher expressions of chondrogenic specific genes (Collagen II, SOX9 and Aggrecan) compared to chondrogenic ADSCs (P = 0.031, 0.010 and 0.013). Grossly, the treated knee joints showed regenerated de novo cartilages within 6 weeks post-treatment. On the International Cartilage Repair Society grade scores, chondrogenically induced ADSCs and BMSCs groups had significantly lower scores than controls (P = 0.0001 and 0.0001). Fluorescence of the tracking dye (PKH26) in the injected cells showed that they had populated the damaged area of cartilage. Histological staining revealed loosely packed matrixes of de novo cartilages and immunostaining demonstrated the presence of cartilage specific proteins, Collagen II and SOX9.

**Conclusion:**

Autologous chondrogenically induced ADSCs and BMSCs could be promising cell sources for cartilage regeneration in osteoarthritis.

## Introduction

On-going findings indicate that stem cell therapy holds promise as a therapeutic option for many diseases. Among other joint degenerative diseases, the treatment of pathologies in cartilage has posed important unmet challenges to the medical community. Cartilage can be elastic, fibrous or hyaline. The hyaline (articular) cartilage covers the smooth load-bearing tissues lining the ends of long bones within the synovial joints [1]. Articular cartilage functions as a nearly frictionless load-bearing surface in diarthrodial joints, withstanding loads of several times body weight for decades as long as it remains healthy [2]. The unique function and properties of cartilage are provided by their tissue's extracellular matrix which is maintained by a population of cells known as chondrocytes (>5% by volume). Because of its small volume of chondrocytes, as well as aneural, avascular and lack of undifferentiated cells properties, cartilage exhibits little to no intrinsic repair capabilities in response to injury or disease [1] [2]. Osteoarthritis (OA), the most common degenerative joint disease comprises of a heterogeneous group of syndrome that affects all joint tissues; characterized by the degeneration of articular cartilages with loss of matrix, formation of fissures and complete loss of the cartilage surface [3]. Traditional efforts to treat articular cartilage damage include joint lavage, tissue debridement, and microfracture of the subchondral bone; abrasion arthroplasty or the transplantation of autologous or allogeneic osteochondral grafts [3] [4] [5]. Although, some of these procedures have yielded promising clinical results, they are generally not applicable to large degenerative diseases such as osteoarthritis [6].

In recent years, there has been a growing emphasis on the use of undifferentiated progenitor cells for tissue engineering, owing to their ability to expand in culture and to differentiate into multiple cell lineages when cultured under specific growth conditions [7] [8] [9] [10]. Owing to these characteristics, adult stem cells from different tissues have been used in various focal cartilage regenerations [11] [12] [13]. We had earlier shown that a single dose of intraarticular injection of autologous bone marrow stem cells (BMSC) could retard the progressive destruction of cartilage in OA sheep model (8). With the recent plethora of interest to adipose stem cells (ADSC) owing to their abundance and easy harvest, it was included in the present study. Both BMSC and ADSC have shown significant chondrogenic potentials for use in tissue engineering approaches [11] [12]. As the field of cellular transplantation matures, methodologies are needed to longitudinally track and evaluate the functional effect of transplanted cells, thus ascertaining the homing of the injected cells. Among the many tracking agents that can be used is PKH26 dye. This red fluorescence cell tracker was developed by (Horan and Slezak 1989) [14] and can be easily detected by conventional fluorescence microscopy and stably incorporates into the cell membrane, allowing for proliferation assessment [15].

Our main objective is to treat OA with cell based therapy. The specific objectives include: firstly to isolate, culture and differentiate ADSCs and BMSCs into chondrocytes. Secondly, to compare the effectiveness of chondrogenically induced ADSCs and BMSCs in treating surgically induced osteoarthritis in sheep model; thirdly to track the induced cells with PKH26 dye after intraarticular injections.

## Material and Methods

### Ethics Statement

This study was carried out in strict observation with the recommendation of ACUC international. Ethics approval was granted by Universiti Kebangsaan Malaysia (UKM) Animal Ethics Committee (PP/TEC/RUSZYMAH/25-NOV/342-DEC-2010-JUN-2012) and Universiti Putra Malaysia (UPM) Animal Ethics Committee (RUJ: ACUC 07R6/JULY 07-DEC 09). All surgery was performed under Xylazine and Ketamine anesthesia; and all efforts were made to minimize pain using Tramadol analgesic.

### Study Design

Un-castrated male sheep (*Siamese long tail cross*) aged 1–2 years, and weighing 20–25 kg (n = 18) comprising of 6 control, 6 ADSC and 6 BMSC confirmed to be healthy were used. The selections to the groups were done randomly and they were housed in pen with slatted floor at a density of 3 animals per pen. The design was to have arthroscopy evaluation of the right knee joint (week 0) in all groups to rule out any pre-existing chondral lesion before surgical operations at (week 1) leading to OA inductions (week 4–6). Then, the second set of arthroscopy was to reveal any induced lesion. Cells were harvested before and during operations, proliferated, labeled with PKH26 (week 1–3) and induced to chondrogenic lineage (week 4–6) then, intraarticulary injected by week 7. Six weeks afterwards, sheep were euthanized and joint samples examined for cartilage regeneration.

### Arthroscopy Evaluations

This procedure was conducted with the aid of an arthroscope (Stryker^R^ Endoscopy Santa Clara CA.). Briefly, a lateral parapatelar skin incision was made proximal to the patella, through which the scope was inserted into the stifle joint. With careful navigation, it was guided through the trochlear groove, the medial and lateral condoyle, then to the tibia plateau. This was performed before surgical operations (week 0) on the knee to ascertain any pre-existing chondral lesions. It was subsequently performed after the full induction of OA (week 6) to visualize the extent of degenerative changes and inflammation developed within the various knee regions; before the injection of chondrogenic induced cell treatments.

### Osteoarthritis Induction

The surgical protocol was conducted according to our previously optimized method [16]. Sheep were sedated with intravenous (IV) xylazine (0.1 mg/ kg) and induced with IV Ketamine (7 mg/kg). Following intubation sheep were ventilated and maintained on isoflurane (1.5%) in oxygen. Analgesic consisted of IV tramadol 2 mg/kg, intra-operatively and repeated 6–8 hourly post operatively for 2days. Prophylactic antibiotics consisted of amoxicillin 20 mg/kg. A medial parapatelar skin incision was made beginning at a level 2 cm proximal to the patella (P) and extending to the level of the tibial plateau (TP). Subcutaneous tissue was incised, and the lateral fascia was separated from the joint capsule for 1 cm in either direction away from the incision. The joint capsule were incised and the patella was subluxated laterally to expose the trochlear groove, medial and lateral condyles of the distal femur. Anterior cruciate ligament (ACL) removal was performed by first excising its attachment on the medial aspect of the lateral femoral condyle (LFC). The proximal attachment is brought forward and the entire ligament was excised from its tibial attachment. The knee joint was moved in a drawer test to ensure that the entire cruciate ligament had been excised. The medial meniscus was removed by sharp excision. The caudal horn of the meniscus was grasped with a hemostat and its lateral attachment was excised from its tibial attachment. Working from caudal to lateral, then cranial, the meniscus was completely removed. The joint was closed using Vicryl sutures 3.0 and 2.0 (Ethicon Inc. USA) for the knee capsules and muscles respectively and 1.0 for the skins. Meloxicam 0.2 mg/kg was administered, and repeated once daily for 3 days. Following extubation, sheep was recovered in its pen and monitored daily for inappetence and wound dehiscence until suture removal, 7 days post-operatively.

### Sheep Exercise

At the end of three weeks recovery period from surgical injuries, sheep underwent exercise conducted in a confined concrete track of 25 meters long and 1 meter wide, running to and fro twice to complete a 100 meters distance. This lasted for three weeks to increase joint contact stress at the operated knee. After the exercise, they were allowed free movement within a pen of 4×4 m^2^.

### Harvest of Adipose Tissue

Adipose tissue was harvested from the right infra patella fat pad during the surgical resections at week 1. Briefly, a medial parapatelar skin incision was made beginning at level 2 cm proximal to the patella and extending to the level of tibial plateau. The medial aspect of the vastus medialis and the joint capsule were incised and the patella was luxated laterally to expose the knee fat pad. Ten milliliters of fat pad were harvested and kept at +4°C until further processing.

### Harvest of Bone Marrow

After scrubbing the skin area covering the iliac spine and sedation prior to operation, a mini incision was made at the most lateral region. 10 mLs of bone marrow was harvested after, with the aid of a trocar (Cardinal Health Inc. USA) and 50 mls syringe (Cringe Malaysia) containing heparin 1×10^3^ units and was kept at +4°C until further processing.

### Isolation of ADSC and BMSC

All samples were processed 6–12 hr after collection. Adipose tissue was minced with a surgical blade (*CE* OEM France Inox) to about 2 mm thick before digestion with an equal volume of 0.6% collagenase II (Gibco USA) in an orbital incubator (Stuart scientific UK) at 37°C, 21 g-force for 2 hr. The digest was filtered with a cell strainer of 100 µm pore (Orange Scientific). The filtrate was then centrifuged (CR3i) to a 4724 g-force for 5 mins at 37°C and the pellet washed with PBS (Sigma USA) twice and basal medium before culture. Bone marrow was isolated and processed using the Ficoll-Paque method (Sigma USA) (16).

### Monolayer Cell Culture

Following the isolation of ADSC and BMSC, they were cultured in 6 well plates (Corning Incorporated USA) with Dulbecco's Modified Eagles Medium/F12 (D-MEM/F12)+10% fetal bovine serum medium (Gibco USA), in a Galaxy^R^ CO_2_ incubator (RS Biotech) at humidified atmosphere of 95% O_2_, 5% CO_2_ and 37°C. After the initial 3 days culture which is necessary for cells to attach; medium was changed every 2 days until the cells were about 90% confluence. They were trypsinized with trypsin-EDTA (Sigma USA), and passaged to T-75 cm^2^ flask (Corning Incorporated USA) at a density of 5×10^5^ cells for an average of four passages. The viability of the cells was calculated using the trypan blue exclusion procedure and the growth kinetics (population doubling time) was evaluated by dividing the total number of cells at the end of the passage by the initial seeding number. These were recorded at every passage.

### Evaluation of Multipotency

Isolated cells from bone marrow and adipose tissue (BMSC and ADSC) were differentiated into the three main cells lineage of mesoderm origin namely: adipocyte, osteocyte and chondrocyte. For the adipogenic inductions, a formula by Buinn Wickham et al [17] was used with modifications. This comprised of Dulbecco's Modified Eagle Media F-10 supplemented with 3% foetal bovine serum, 100 units/mL penicillin, 100 ug/mL streptomycin, 15 mmol/L HEPES buffer solution (pH 7.4), biotin (33 um, Sigma), Calcium Pantothenate (17 um), human recombinant insulin (100 nmol/L), 3-isobutyl-1-methylaxanthine (0.25 mmol/L), and Rosiglitazone (1 umol). Oil red staining was used to evaluate the adipogenic differentiation for lipid deposition. Briefly, the adipogenic cultures were fixed by removing the culture media and gently rinsing the flask with 10 ml sterile DPBS. Then 10 ml, 10% formalin was added and incubated for 30–60 mins at room temperature. The working stock solution of Oil Red O was prepared and the staining was done according to the protocols. After this, sample was washed with tap water and viewed on phase contrast microscope. Lipid appears red and nuclei appear blue.

The osteogenic induction was done using an optimised formula from our lab that comprised of FD+10% FBS medium containing Hans FD/F12, Antibiotic- antimycotic, Glutamax, Vitamin C, Herpes 2.4 mg/ml, and 10% foetal bovine solution (FBS). Other components include dexamethasone, β-glycerophoshate and ascorbic acid-2- phosphate. Medium was changed every three days and the induction period lasted for 21 days. Calcium deposition in the osteogenic differentiated cells was evaluated using Alizarin Red Staining. Alizarin Red was done by fixing the cultures with cold ethanol for 1 hour and rinsed. Fixed cultures were incubated with Alizarin red for I hour, then with boric acid buffer before counterstaining with haematoxylin. The whole stain was evaluated using bright field microscopy (Olympus-CK40).

Chondrogenic induction was done for both BMSCs and ADSCs using an optimised formula from our lab as explained in the section below. Evaluation of chondrogenesis was done using Toluidine blue staining (Gainland UK) to detect the proteoglycans formation and matrix accumulations. Briefly, sections were deparaffinised and dehydrated in distilled water; stained in Toluidine blue working solution for 2–3 minutes before washing in distilled water. Stained sections were dehydrated through (95 and 100) % alcohol; cleared with xylene before mount using DPX fluid (Gibco USA) for microscopic examination to detect extracellular matrix and proteoglycans formation. Cytoplasm stains blue, nuclear materials stain dark blue and cartilage ECM/mast cells stain purple.

### PKH26 Staining

Cells were stained following an optimized protocol in our earlier study [18]. Briefly, 2×10^7^cells were trypsinized and counted via trypan blue (Gibco USA) exclusion using haemocytometer (Neubauer Improved-Germany) for total cell number and viability before staining. ADSCs were stained with 2 µmol and BMSCs with 8 µmol of PKH26 dye in 15 ml polypropylene tubes. Cells were monitored with light microscope (Olympus-CK40) and live imaging fluorescence microscope (Nikon-Eclipse Ti). After sacrifice, the visible regenerated areas on the treated knee joints were analyzed for the presence of the tissue-engineered chondrocytes with the Confocal Microscope (NIKON -AIR).

### Chondrogenic Induction

The chondrogenic medium was prepared following our optimized formula [19], by the addition of Dulbecco's Modified Eagles Medium/F12 (D-MEM/F12) (Gibco USA) 93.5%, Fetal bovine serum and Glutamax (Gibco USA) 1%, Antibiotic antimycotic (Gibco USA) 1%, Vitamin C (Sigma USA) 1%, Insulin transferring selenium (Gibco USA) 1%, IGF-1 (Invitrogen Inc.) 50 ng/ml, Ascorbic acid-2-phosphate (Sigma USA) 50 ug/ml, L- Proline (Sigma USA) 40 ng/ml, Dexamethasone (Invitrogen Inc.) 100 nM and Tissue growth factor-beta 3 (TGF-β3) (Invitrogen Inc.) 10 ng/ml. Immediately after PKH26 staining, cells were cultured in the prepared chondrogenic medium. Medium was changed every 3–4 days and the induction period lasted for three weeks.

### Quantitative RT-PCR Assay

Total ribonucleic acid (RNA) from the samples of BMSC and ADSC at early P0 cultures (1week post isolation) and after induction to chondrocytes (6 weeks post isolation), was extracted by dissolution in trizol reagent (Gibco BRL, USA). Complementary DNA was synthesized using the iscript (BIO-RAD) and analyzed for gene expression using the iQ SYBR^R^ green super mix (BIO-RAD) on MyiQ single colour Real Time polymerase chain reaction (RT-PCR) detection system (BIO-RAD). Primers (Biobasic Canada.) were used to determine transcript levels in triplicate for a housekeeping gene glyceraldehydes-3-phosphate dehydrogenase (GAPDH) (F: 5^I^ –ctggtgctgagtacgtggtg–3^I^, R: 5^I^ –cgtcagcagaaggtgcagag–3^I^) and four different genes of interest namely: Collagen type II (Col II) (F: 5^I^ –cctcaagaaggctctgctca–3^I^, R: 5^I^ –atgtcaatgatggggagacg–3^I^), Aggrecan Core Protein (Agg) (F: 5^I^ –taggtggcgaggaagacatc–3^I^, R: 5^I^ –aaacgtgaaaggctcctcag–3^I^), SRY (sex determining region y)-box 9 (SOX9 genes) (F: 5^I^ –tgaatctcctggaccccttc–3^I^, R: 5^I^ –cttgtcctcctcgctctcct–3^I^) and collagen type I (Col I) (F: 5^I^ –cggctcctgctcctcttagcg–3^I^, R: 5^I^ –ctgtacgcaggtgactggtg–3^I^). Data was analyzed by calculating the fold differences in gene expressions of the differentiated cells compared to undifferentiated cells after they were normalized to their own GAPDH value.

### Intraarticular Injections of Induced BMSCs and ADSCs

On the 7th weeks (1 week after OA inductions), intraarticular injections were done according to our previously optimized method [16]. Briefly the cells were trypsinized, washed with PBS (Gibco USA) and culture medium, resuspended in culture medium at a density of 4×10^6^cells/ml in a 5 ml cell injection. Sheep was anesthetized with intravenous (IV) xylazine (0.1 mg/kg) and Ketamine (7 mg/kg); then placed on lateral recumbence. Using the para ligamentous technique, an18-guage needle (Cringe Malaysia) was inserted posterior to the medial edge of the patellar ligament, through the triangle formed by the epicondyle of the femur, the tibia plateau and the notch at their junction. Cell suspension was injected into the operated knee of the BMSC and ADSC samples after aspiration of synovial fluid, while the controls received an equal volume of culture medium. The joint was repeatedly flexed and extended for the dispersal of the injected cells.

### International Cartilage Repair Society (ICRS) Evaluation

At the end of six weeks post intraarticular injection, sheep were euthanized. The ICRS scale of OA assessment was used to evaluate the degenerations and cartilage repairs in both the control and test sheep. It has five score grades namely: Grade 0 – Normal; Grade 1 - Soft indentation superficial fissures and cracks; Grade 2 - Lesions extending down to <50% of cartilage depth; Grade 3 - Cartilage defects extending down >50% of cartilage depth to calcified layer; Grade 4 - Severely abnormal, extending down through the subchondral bone. The articular cartilage lesion was graded by two independent blinded orthopaedic scorers.

### Haematoxylin and Eosin

Sections of 10 um thickness were stained with Haematoxylin and Eosin to assess cell morphology. Samples from the visible regenerated portions of the patella femoral groove (PFG) on both the treated samples and the degenerated part on the control sheep were deparaffinized with xylene and dehydrated with ethyl alcohol (Essen-Haus Sdn. Bhd). They were stained with Hematoxylin Harris (DAKO, Glostrup Denmark) and counter stained with Eosin (DAKO, Glostrup Denmark). Slides were viewed by the light microscope after dehydration with alcohol and xylene (VWR International LTD).

### Safranin O Stain

Slides of 10 um thickness from the patella (P) on both the treated samples and the degenerated part of P on the control sheep were stained with Weigert's iron haematoxylin (Sigma USA) working stock solution. They were further stained with fast green solution (Dako, Denmark), and counter stained with Safranin O solution (Stain pur), before mount using DPX fluid (Gibco USA) for microscopic examination to detect proteoglycan accumulations.

### Immunohistochemistry

Sections of 10 um from the regenerated portions of PFG and a subsequent 10 um section of healthy cartilage from the un-operated PFG were pretreated with Tris-buffered saline (TBS) (Sigma, Inc) for one minute. Antibodies were retrieved using pH 9 buffer (DAKO, USA) in boiling water for 20–40 min at 95°C. Antibody binding was blocked by incubation in 10% normal goat serum (Gibco, USA) at 37°C for 30 min. Rabbit anti-sheep Col II, SOX9 and Col I polyclonal antibodies (iDNA Biotechnology Malaysia) were applied as primary antibody at 4°C for 14–18 hr. Sections were incubated with the secondary antibody, sheep anti-rabbit immunoglobin tagged with green fluorescence (iDNA Biotechnology Malaysia) for 1–2 hr and counter stained with 4, 6-diamino-2-phenylindole (DAPI) (Sigma, Inc.). Col II, SOX9 and Col I were detected using confocal microscopy. The native articular cartilage served as the positive control for Col II and SOX9, while the fibrous cartilage served as the positive control for Col I. The regenerated cartilage sections without primary antibody served as negative control for all.

### Statistical Analysis

Data were presented as mean ± standard error of mean (SEM) of sample size. The parametric means which compare the means of two samples or treatments in a normal distribution were analyzed using paired student's t-tests. In the gene analysis, we effectively used each sample as its own control hence the correct rejection of a null hypothesis can become much more likely. The ICRS were done using the Mann Whitney U-test for non-parametric mean. Uncertainties were presented within 95% confidence intervals and all statistical analysis was performed using the version 17.0 of the SPSS software.

## Results

### Arthroscopy and Arthrotomy

**Figure 1:**
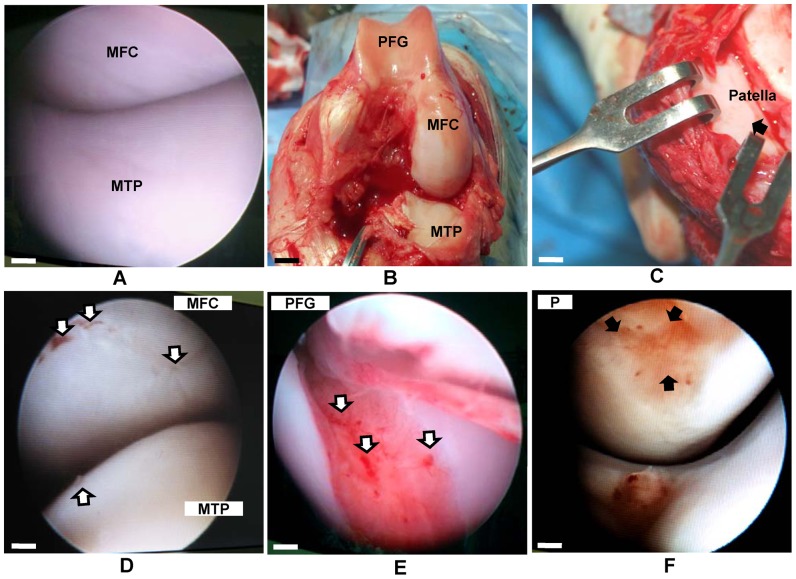
The arthroscopic and arthrotomy representation of the right knee joints before and after surgical inductions. (**a**) The arthroscopic representation of the right knee joints before the surgical operations revealed no chondral lesion on medial femoral condyle (MFC) and medial tibial plateau (MTP). (**b**) The arthrotomy photo during the surgical operations revealed no previous lesions at Patella femoral groove (PFG), medial femoral condyle (MFC), medial tibial plateau (MTP). (**c**) The arthrotomy photo of patella (P) before OA induction revealed no chondral degenerations (black arrow). (**d**) The arthroscopic images after OA inductions at MFC and MTP showed slight indentations and moderate focal lesions (white arrows). (**e**) Arthroscopic images after OA inductions at the PFG revealed severe and prominent chondral erosion (white arrows). (**f**) The arthroscopic images after OA inductions at P, which is the opposite contact of PFG, depicted degenerations of cartilage caused by the frictional contacts. Arthroscopy scale  = 0.5 cm, Arthrotomy  = 1.5 cm.

The arthroscopy examination conducted on the right knee joints (Fig. 1a) before the surgical operations revealed that the target regions medial femoral condyle (MFC) and medial tibial plateau (MTP were free of chondral lesions.The arthrotomy photos taken during the surgical resections (Fig. 1b & c) still revealed that the Patella femoral groove (PFG), medial femoral condyle (MFC), medial tibial plateau (MTP), and patella (P) had no chondral degenerations. Then after OA inductions (Fig. 1d, e & f), the arthroscopic images showed degenerative and inflammatory changes within the various regions of the knee examined. The MFC and MTP showed moderate focal lesions to slight indentations. The lesions at the patella femoral groove (PFG) were severe and prominent. It was not only seen on the groove, but also at the opposite side patella (P), depicting a degeneration of cartilage caused by the frictional contacts between the two surfaces owing to ACL resection.

### Evaluation of Multipotency

**Figure 2:**
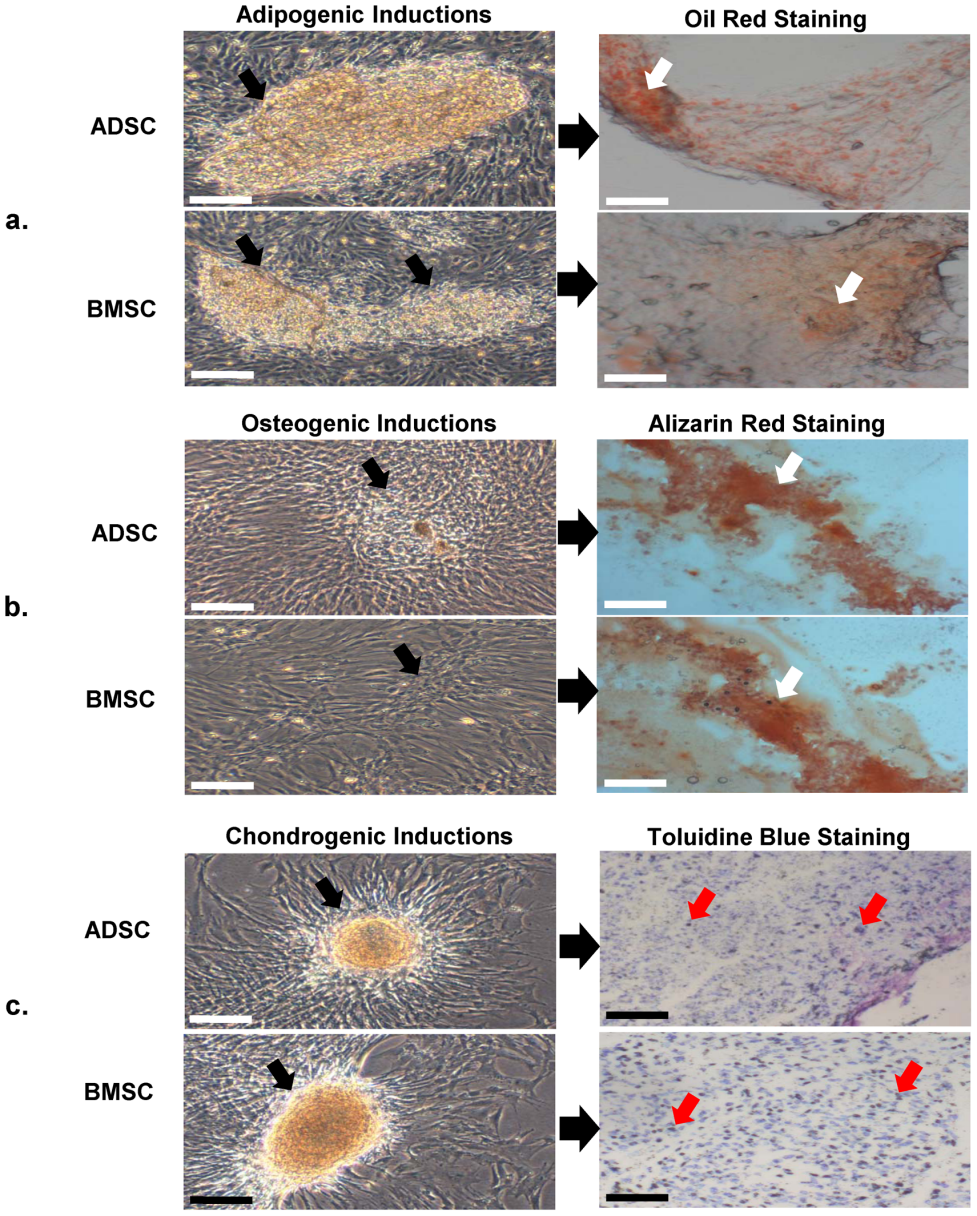
Multipotency evaluations of ADSCs and BMSCs. (**a**) Inverted phase contrast images of the Adipogenic inductions of ADSCs and BMSCs. The image depicted the collection of fat droplets in clusters (black arrows) from day 4 of the inductions process. The oil red staining showed that lipids formed on both induced cell samples, picked up red stain (white arrow) showing their positivity to adipogenic lineage. Scale  = 70 µm. (**b**) Inverted phase contrast images of the osteogenic inductions of ADSCs and BMSCs. The picture revealed similar clustered mineralization on both cell samples from day 4 of the inductions process. The evaluation of the induced ADSCs and BMSCs with alizarin red staining demonstrated some mineralization activities (white arrow) showing their commitment to osteogenic lineage. Scale  = 70 µm. (**c**) Inverted phase contrast images of the chondrogenic inductions of ADSCs and BMSCs. The picture revealed similar aggregation of the cells in condensations seen in early chondrogenesis from day 4 of the inductions process. The evaluation of the induced ADSCs and BMSCs with toluidine blue staining demonstrated that the condensed matrixes picked up the blue stain (red arrows), showing their positivity to chondrogenic lineage. Scale  = 70 µm.

Both cell samples from ADSCs and BMSCs demonstrated their capacities for adipogenic differentiations. From the third day of induction, lipid droplets were noticed within the cells and as the induction progressed, the formed droplets clumped together in clusters of shiny oily appearances, though more in ADSCs culture (Fig. 2a). At the end of the induction period, Oil red staining was used to confirm the lipid depositions as seen in early adipogenesis.

Osteogenic differentiation caused both cells to form stratified-like cluster of cells from the fifth day. These clusters, which are typical characters of early mineralization and calcium deposition seen in osteoblasts differentiation multiplied in number throughout the flask from day twelve (Fig. 2b). ADSCs seem to have more of the cluster formations. Alizarin Red stain was used to confirm the presence of calcium deposition and both cells picked up the dye.

On chondrogenic evaluations, both cells showed signs of cell aggregation and matrix deposition from day three, though more prominent in BMSCs culture. As the induction progressed, the aggregation of cells turned into pockets of nodules with cell sheets folding them (Fig. 2c). This is typical of condensation seen in early chondrogenesis. Toluidine blue stain confirmed the extracellular matrix and proteoglycans formation on both cells.

### Monolayer Cultures

**Figure 3:**
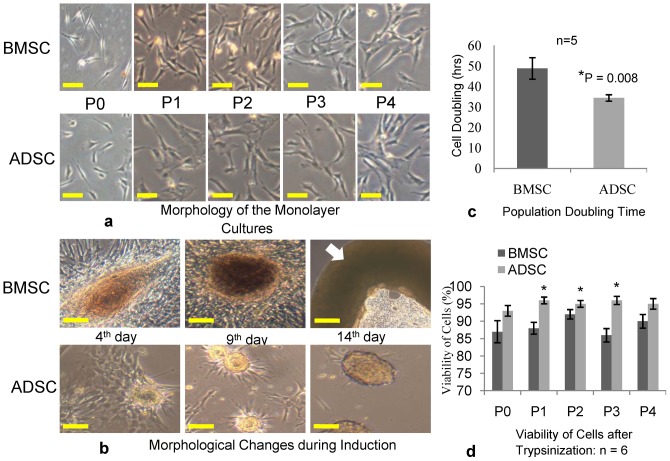
Monolayer analysis of BMSCs and ADSCs. (**a**) The morphological images of the BMSCs and ADSCs from P0 to P4. BMSC looked more spindle upon isolation and early attachment, while ADSCs were broader (P0-P1). Both cell samples attain the same size and shape as the passage progressed from P2–P4 showing more mesenchymal-like structure. Scale bar represents 35 µm. (**b**) The Morphological changes of the cells during chondrogenic differentiation. BMSCs formed the aggregates of cartilage in a film-like sheet, more readily than ADSCs. The aggregates and sheet clumped together (white arrow) to form a firm cartilage structure by 3rd week. ADSCs split its formed aggregates and dispersed on the medium. Scale represents 35 µm. (**c**) The Population doubling time (PDT) of the cell samples during culture. ADSCs were 34.4±1.6 hrs, while that of BMSCs were 48.8±5.3 hrs. ADSCs had a significantly higher growth rate compared to the BMSCs with P value of 0.008. (**d**) The viability of cells after trypsinization, at the end of each passage. From P0–P4, BMSCs had mean viabilities of (87, 88, 92, 86, and 90); while ADSCs had (93, 96, 95, 96, and 95) respectively. ADSCs had higher viabilities at each passage compare to BMSCs but were significantly higher at P1, P2 and P3; P<0.05.

After the isolation and culture of stem cells from the same volume of tissues (each 10 mLs of adipose or bone marrow), ADSCs showed more adhesion to the flask and proliferation by the second day. At the fourth day, the attached ADSCs were replicating faster compared to BMSCs. ADSCs were the first to reach confluence at passage zero (P0) in 6 well plates. At P0 and P1, BMSCs look more spindles and smaller in shape than ADSCs. As the passage increased from P2 to P4, both cells get even and looked alike with no differences in shapes and sizes Fig. 3a. Subsequently, the cells morphology during chondrogenesis revealed that BMSCs formed aggregates and matrixes of proteoglycans more readily than ADSCs. As the induction progressed, BMSCs aggregate-matrixes and the cell sheets clumped together to become a firm cartilage structure by the 3rd week of induction. ADSCs on the other hand, did formed aggregates of chondrogenic matrix and proteoglycans but with minimal sheet. From the third day of induction, the formed aggregates split and dispersed on the medium Fig. 3b.

### Growth Kinetics and Viability

After the initial P0 cultures, cells were seeded at low density of 5000 cell/cm^2^ in 75 cm^2^ culture flask. It was observed that ADSCs attained confluence faster than BMSCs from P1 to P4. The proliferation rate of ADSCs was 34.4±1.6 hrs and that of the BMSCs was 48.8±5.3 hrs; thus ADSCs had significantly higher growth rate compared to BMSC p<0.05 Fig. 3c. At every trypsinization, BMSCs detached faster than ADSCs using equal volumes of 0.25 M concentration of trypsin EDTA. The viability of cells after trypsinization showed that ADSCs had higher viability at all the passages compared to BMSCs. At the 95% confidence limit, ADSCs viabilities were significantly higher at P1, P2 and P3, Fig. 3d.

### Chondrogenic Induction and Gene Expressions

**Figure 4:**
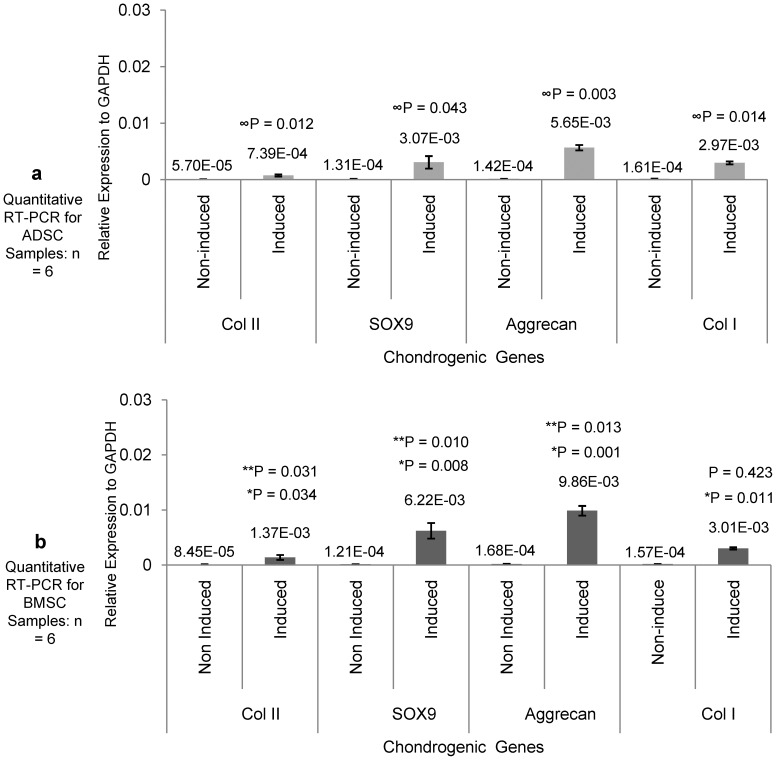
Gene expression analysis of ADSCs and BMSCs. (**a**) Comparison of the gene expressions of chondrogenically induced ADSCs to the uninduced. After 21 days of induction, the induced samples had significantly higher expressions of Col II, SOX9, Aggrecan and Col I genes in fold increases of (12.96, 23.44, 44.36 and 18.44) respectively compare to the uninduced (week 1 post-isolation) cells, with P values (∞) respectively (P<0.05). (**b**) Comparison of the gene expressions of chondrogenically induced BMSCs to the uninduced. After 21 days of induction, the induced samples had significantly higher expressions of Col II, SOX9, Aggrecan and Col I genes in fold increases of (16.21, 51.40, 120.33 and 19.17) respectively compare to the uninduced (week 1 post-isolation) cells, with P values (*) respectively (P<0.05). Comparing the Induced ADSCs and Induced BMSCs, BMSCs had significantly higher expressions of Col II, Aggrecan and SOX9 genes in fold increases of (1.85, 2.03 and 5.31) respectively with P values (**) (p<0.05). There was no significance difference on Col I expression.

After the normalization with their GAPDH, the expressions of the specific genes for chondrocytes on the induced samples of BMSCs and ADSCs were higher compared to the uninduced samples. Chondrogenically induced ADSCs had 12.96, 23.44, 44.36 and 18.44 folds increases of Col II, SOX9, Aggrecan core protein and Col I genes respectively Fig. 4a, while the chondrogenically induced BMSCs had 16.21, 51.40, 120.33 and 19.17 fold increases respectively which were significant at 95% confidence limit, Fig. 4b. Compared to ADSCs, BMSCs had significantly higher expressions (1.85, 2.03 and 5.31) of Col II, Aggrecan and SOX9 genes respectively but not on Col I (p<0.05).

### Gross Evaluations

**Figure 5:**
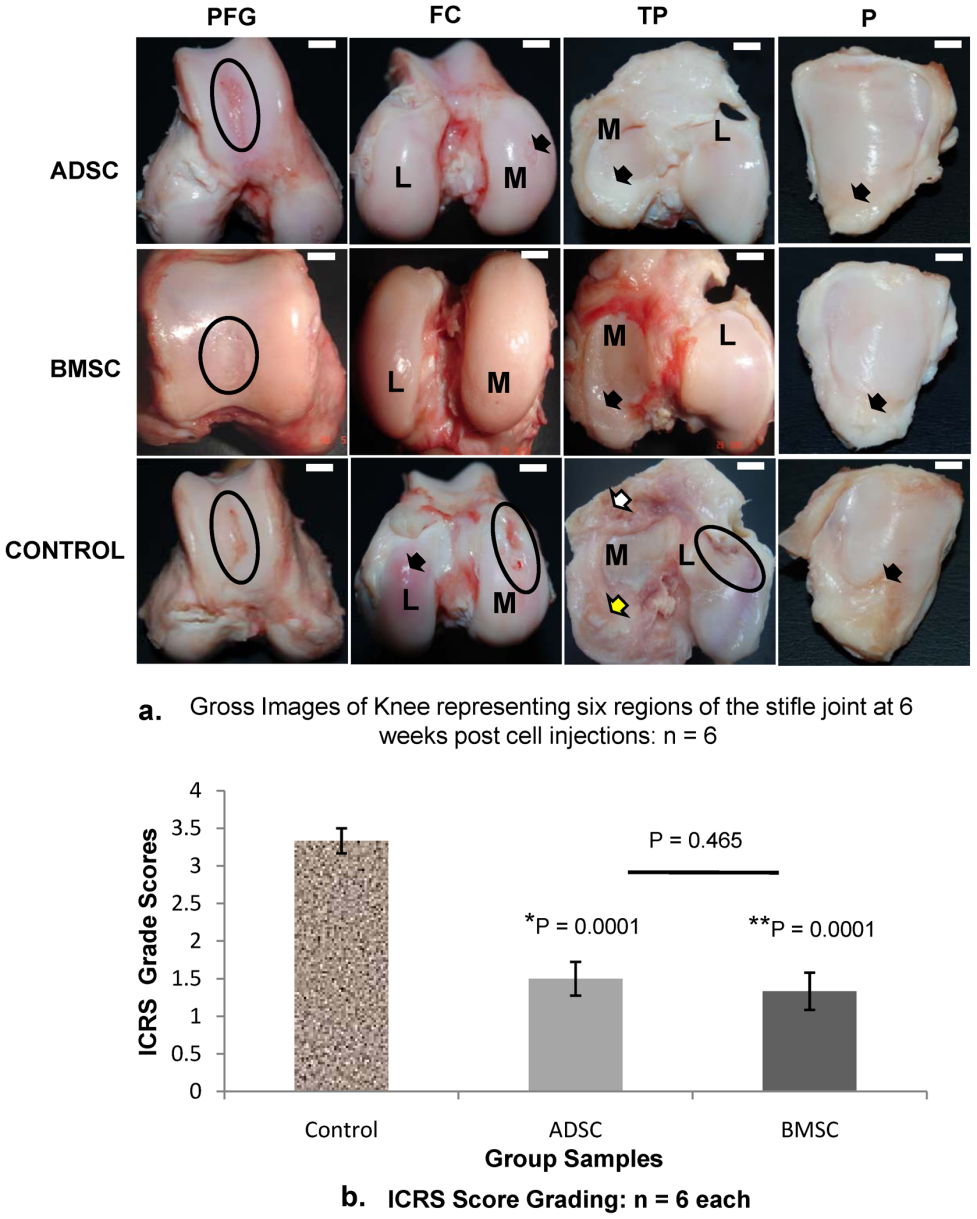
The gross evaluations of the right knee joint samples. (**a**) The gross images of knee representing the six regions. Patella femoral groove (PFG), medial femoral condyle (MFC), lateral femoral condyle (LFC), medial tibia plateau (MTP), lateral tibia plateau (LTP) and patella (P) were used as reference points. The treated knees showed regenerations. At PFG, the regenerated cartilages had unique morphological appearances different from the native (black ring); the control still retained severe cartilage degeneration (black ring). At MFC, the ADSCs still retain a slight focal defect (black arrow); BMSCs had no sign of defect, while the control retained severe cartilage degeneration (black ring). At LFC, there were no lesions on the treated knees, but the control had reduced cartilage thickness (black arrow). At MTP, both treated samples revealed structural appearances of a crescent regenerating meniscus-like cartilage (black arrow), but none at the control. There is no evidence of meniscus regeneration on the medial tibia plateau (yellow arrow). There is also a remnant of resected spur bone formed at the anterior region of the medial tibia plateau (white arrow), which is a supportive mechanism seen in severe OA. At the LTP, there were no conspicuous lesions at the treated samples but the control retained a severe degeneration (black ring). At P, the control presented worse degeneration compared to either of the ADSCs or BMSCs (black arrows). (M =  medial, and L =  lateral). Scale represents 1.5 cm. (**b**) Combined gross and histological ICRS grading of the right knee joints. The control sheep scored a mean grade of 3.33±0.2, while the test groups ADSCs and BMSCs scored 1.5±0.2 and 1.3±0.3 respectively. Both treated sheep samples had significantly higher grades (*) and (**) respectively compared to the control P<0.05. BMSCs treated sheep had better grade score than ADSCs, but was not significant P = 0.465.

The treated joints had varying degrees of regenerated cartilages. The regenerations at patella-femoral groove (PFG) were more prominent and had a distinct morphological appearance. They were different in appearance compare to the native cartilage and were not present on controls Fig. 5a. The medial femoral condyles (MFC) of the treated sheep had decreased cartilage degenerations and possible cartilage regeneration. They were better that the control samples which showed deep cartilage degeneration. There were no much differences from samples at the lateral femoral condyle (LFC), except for the controls that revealed swollen reduced cartilage thickness which will likely deteriorate with time Fig. 5a. At the medial tibia plateau (MTP), the treated samples had visible signs of cartilage regeneration and most prominently, the formation of a crescent structure-like meniscus in attempt to replace the resected meniscus. The control samples had no signs of meniscus regeneration; instead there was a remnant of spur bone formed at the anterior region of the medial tibia plateau (white arrow), which is a supportive formation seen in severe OA development. There were reduced signs of cartilage degeneration at the lateral tibia plateau (LTP) for the treated sheep samples, but deep cartilage defects on the control samples Fig. 5a. The patella (P) showed reduced cartilage degeneration on the treated samples, while the controls revealed remarkable deep cartilage defects Fig. 5a. Generally, the treated sheep had varying degrees of regenerations, but the control sheep had little evidence of cartilage regeneration and the area of degeneration were prominent in all the regions. On the ICRS grade scale, the control sheep scored a mean grade of 3.3±0.2, while the test groups ADSCs and BMSCs scored 1.5±0.2 and 1.3±0.3 respectively Fig. 5b. Using the 95% confidence interval estimation, both treated knee were highly significant compared to the control with P values of 0.0001 and 0.0001 respectively. BMSCs treated sheep had better scores of ICRS than ADSCs but was not significant P = 0.465.

### Fluorescence of PKH26 Dye

**Figure 6:**
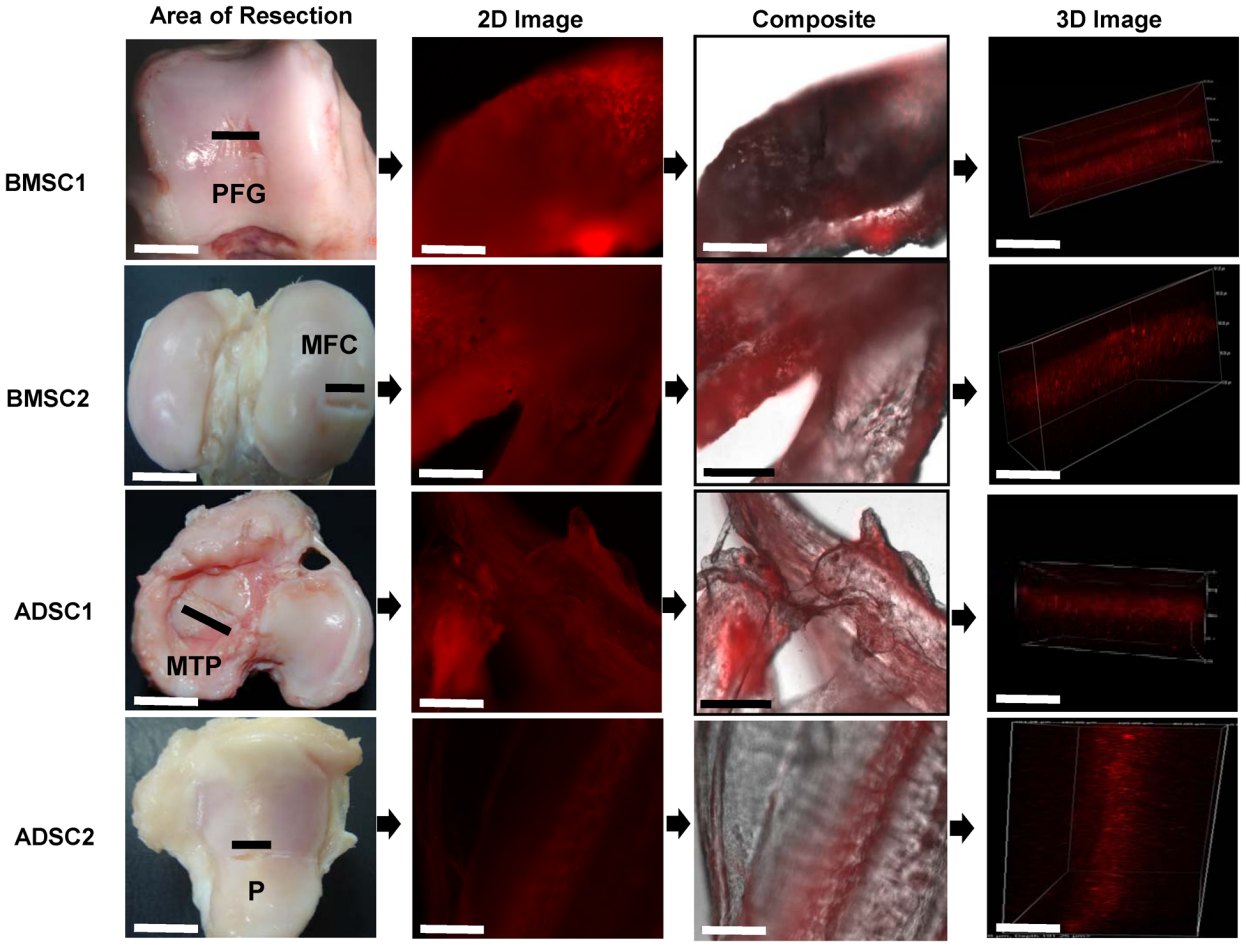
Fluorescence evaluations of PKH26 dye on the resected regenerated cartilages. Samples were taken from PFG and MFC of the BMSCs samples; then from MTP and P of the ADSCs samples. PKH26 fluorescence was shown in 2D, the composite images and 3D images. The composite and 3D confocal images revealed the integration and arrangements of the labeled chondrogenically induced cells. The fluorescence of the dye proved the participation of the injected cells in the cartilage regeneration. Scale: gross images  = 1.5 cm and microscopic images  = 35 µm.

The monolayer pictures of the labeled cells showed the cytoplasm stained with PKH26. Fluorescence microscopic analysis of the resected portions of neo-cartilages from the treated right knee joints revealed red fluorescence of PKH26 on the various regions with visible regenerations Fig. 6. These include: PFG, MFC, MTP and P. The composite images from these regions which comprised a phase contrast and the fluorescence image depicted the possible interactions of the labelled cells with the native cartilage. With the confocal microscope, three-dimensional (3-D) layered arrangements of integration of the neo cartilages with the native could be appreciated. The fluorescence confirmed that the injected cells were on the articular surfaces of the treated joints.

### Histological Evaluation

**Figure 7:**
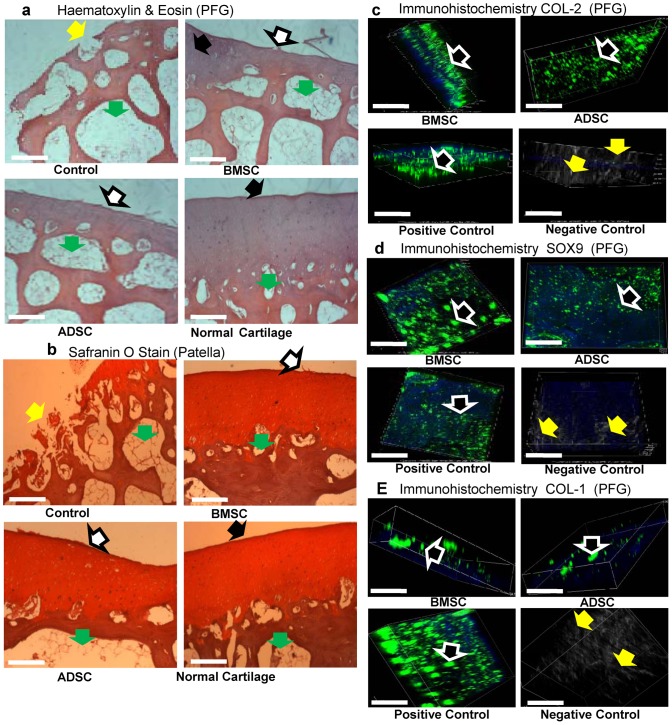
Histological and immunohistochemical evaluations of the right knee joints. (**a**) Haematoxylin and Eosin stain of the right knee joint PFG samples. Yellow arrow points at the exposed area of cartilage, leading to subchondral bone (green arrow) of the control knee joints. Treated samples (BMSCs and ADSCs) reflected regenerated engineered cartilages (white arrows) covering the subchondral bone, though not smoothly packed like the native cartilage (black arrow). Scale represents 70 µm. (**b**) Safranin O stains of the right knee joint (patella) samples. The regenerated cartilage on both treated knee (BMSCs and ADSCs) stained positive (white arrow). They were homogenous to the histochemical properties of accumulated proteoglycans revealed via Safranin O, but not smooth as the native cartilage (black arrow). Yellow arrow points at the degenerated area of cartilage on the control; green arrow, the subchondral bone. Scale represents 70 µm. (**c**) Immunohistochemistry images of the right knee joint (PFG) samples. Collagen type II protein staining for both treated samples (BMSCs and ADSCs) demonstrated positive staining within the ground tissues and were visible throughout the matrixes, evidenced by the green fluorescence tagged with the secondary antibody (black arrow). The native articular cartilage served as positive control (black arrow). The absence of green fluorescence on the ground tissue of the engineered cartilages without the primary antibody (yellow arrow) served as the negative control. Scale represents 35 µm. (**d**) Immunohistochemistry images of the right knee joint (PFG) samples. SOX9 staining for BMSCs and ADSCs demonstrated positivity within the ground tissues and were visible throughout the matrixes by the green fluorescence tagged with the secondary antibody (black arrow). The native articular cartilage served as positive control (black arrow), while the absence of green fluorescence on the ground tissue of the engineered cartilages without the primary antibody (yellow arrow) served as the negative control. Scale represents 35 µm. (**e**) Immunohistochemistry images of the right knee joint (PFG) samples. Collagen type I staining for BMSCs and ADSCs demonstrated diminished positivity within the ground tissues. This occurred mainly as scattered clusters of the green fluorescence tagged with the secondary antibody (black arrow). The fibrous cartilage served as positive control (black arrow), while the absence of green fluorescence on the ground tissue of the engineered cartilages without the primary antibody (yellow arrow) served as the negative control. Scale represents 35 µm. **Note**: The authors wish to state that a superimposed histological image of Safranin O and PKH26 dye or the immuno signals with PKH26 dye would have made better proves of the presence of the injected cells, but owing to technical challenges with these processes and the dye stability, it was not accomplished.

The tissue-engineered cartilages from the visible regenerated portions of the PFG on both treated sheep samples showed slight signs of lacunae and cartilage isolated cells on haematoxylin and eosin staining. Though the regenerated cartilages exhibited the stain of well distributed cartilage cells within the basophilic ground substances, they were not smoothly packed like the native cartilage. The controls showed reduced cartilage layer and exposure of the subchondral bone Fig. 7a. The regenerated cartilage from the P on both treated samples stained positive with Safranin O. They were avascular and homogenous in accordance to the accumulated proteoglycans revealed in Safranin O staining. There was no difference in the stains of ADSCs compared to the BMSCs, but the control samples showed deep cartilage defects Fig. 7b. On immunohistochemistry analysis, both treated knee samples from the regenerated portions of PFG demonstrated expressions for Col II (the major oligomeric protein in hyaline cartilage), SOX9 (the main transcription factor for cartilage) and Col 1(a marker of fibrous cartilage) proteins within the ground tissues. Both Col II and SOX9 antigens can be highly visualized throughout the matrix of the engineered cartilages by the green fluorescence of the secondary antibody Fig. 7c and 7d; while the expression of negative marker Col I was scanty present within the ground tissue Fig. 7e.

## Discussions

Cell based therapeutic approaches in tissue engineering and regenerative medicine has highlighted the need for the utilization of the abundant, undifferentiated progenitor cells in various tissues [2]. This study compared adult stem cells from adipose tissue (ADSCs) and bone marrow (BMSCs) that were cultured under the same chondrogenic conditions and duration. Both cell samples exhibited multipotency abilities at least, to the three main cell types of mesoderm origin evaluated, adipocyte, osteocyte and chondrocyte. This was in agreement with some of the earlier reports in pluripotency of adult stem cells from various tissues [1] [2] [11] [17] [20]. Our observation that ADSCs has more cells attached than BMSCs from the second day of culture was a further indication as suggested earlier that, ADSCs had relatively abundance cells available as compared to BMSCs [20] [21] [22] [23]. It had also been reported that ADSCs achieved higher passage number compared to BMSCs before senescence [24] [25] [26]. The trypsinization experience suggested that ADSCs may have secreted more extracellular matrixes for adhesion and needed more time to dissolve after the application of trypsin EDTA. This extra cellular matrix might also have protected their contacts with trypsin EDTA thus, gave them higher viability after trypsinization. This is a new observation from this study and needed more experiments for confirmation as it was consistent in all the passages and with different concentrations of trypsin EDTA (data not shown).

Using the same chondrogenic formula which was necessary for a fair comparison of gene expression and cartilage regeneration from both cells, BMSCs had higher expressions on all the four genes compared to ADSCs. Col II which is the most abundant hyaline cartilage oligomeric matrix protein; SOX9, a major transcription factor; aggrecan, the predominant proteoglycan and Col I, a negative marker to hyaline cartilage [20], [27] were highly expressed by the BMSCs. This was reflected on the nature of cartilages formed and the regeneration that followed on the treated sheep. BMSCs induced better than ADSCs using our induction medium, even though they also had higher expression of the fibrotic marker. Immunohistochemistry showed a high presence of SOX9 transcription protein, Col II together with a diminished presence of Col I protein on both treated knee samples. The presence of Col II throughout the matrix of both samples was as a result of the in vivo three-dimensional interactions of the cells. This confirmed the phenotype of maturing hyaline-like cartilages in contrast with the in vitro gene expressions. The diminished expression of Col I was also due to the same course. Previously in our work, we had shown that cartilage induction in two-dimensional monolayer culture flask expressed less Col II and more Col I with cells retaining fibroblastic morphology. These expressions were changed towards typical Col II and polygonal morphology of hyaline-like chondrocytes, when the cells were implanted in vivo under the skin of nude mice [28].

It was reported that ADSCs have reduced or absent transforming growth factor β (TGF- β) receptor ALK-5 [29], and cell surface marker vascular cell adhesion molecule 1 (CD106) [20] thus, under standard chondrogenic differentiation conditions, typically utilizing TGF- β and dexamethasone, BMSCs have an enhanced potential for chondrogenesis compared to ADSCs [24] [30]. However, other reports suggested that ADSCs had been shown to be more efficiently induced towards chondrogenic lineage by a high dose of bone morphogenetic protein-6 (BMP-6) than by TGF-β or other cocktails [20] [29]. Kim and Im reported that higher concentration of growth factors, specifically fivefold of TGF-β and IGF for ADSCs is required to overcome the chondrogenic differences with BMSCs [21] [31] [32].

The arthroscopic and arthrotomy images of the joint before OA inductions showed that they were free of degenerations. Trauma to the ACL, meniscus and isolated cartilage lesions have been reported to be responsible for osteoarthritis of the joint with time [33]. Our combination of resections and exercise regimen caused severe multifocal cartilage degenerations typical of OA. The chondrogenically induced injected cells elicited varying degrees of regenerations on the test samples compared to the controls as represented by the ICRS grading. This reflected decreased cartilage degenerations and increased regenerations on the treated animal compared to controls. In addition, the regeneration of the meniscus seen in the treated animals only is also a remarkable feature in this study, being that naturally, meniscus is less likely to regenerate. Among the many surgical methods to regenerate cartilage [11] [12] [13] [34] [35] [36] microfracture has been the most favorable [33]. Clinical studies of autologous chondrocytes transplantation generally have reported significant improvements in function [2]. However these techniques retain a high probability of fibrous tissue formation, periosteum flap hypertrophy, cell apoptosis, cartilage degeneration, incomplete hyaline cartilage generation and donor site morbidity [26]. These issues have encouraged studies with injectable chondrogenically induced ADSCs and BMSCs in cartilage regenerations. Our results gave hope for the first time, a prospective clinical regeneration of cartilage that does not involve surgical implantations, autologous chondrocytes, invasiveness and related complications in 6 weeks; only a single dose injection of the appropriate number of induced chondrocyte is required to regenerate full chondral degradations.

The histological evaluation of the engineered cartilage with haematoxylin and eosin at PFG, showed the histoarchitectural characteristics of well distributed chondrocytes within the basophilic ground substances. The arrangement of the condensing cartilage could be seen just as earlier reports [20]. The test samples also stained positive for safranin O at P, but presented rough outer periphery compared to the native cartilage.

In our earlier study, it was found that both uninduced BMSC and chondrogenic induced BMSC could retard the progression of OA, though the chondrogenically induced cells indicated better results [8] [16]. There have been varied reports on probable paracrine effects of the pluripotent stem cells to illicit regenerations [37] [38]. Efforts to clarify the authenticity of our claims from possible regenerations done by migrating cells or secretory vesicles necessitated the labeling of both cell samples to ascertain their homing. PKH26 dye was detected on the resected portions of regenerated cartilages in all 4 regions of the right knee joints. This consolidated our previous and the present claim; and proved that the injected chondrogenically induced cells participated in the regeneration of the osteoarthritic lesions within 6 weeks.

Limitations: Considering that sheep matures at about 18months and osteoarthritis is a disease of the elderly, the first limitation to this study was the age of some of the experimental animals. Due to the difficulty in procuring the desired older samples, these teenage sheep were used in our study. Secondly, it was our view to evaluate the dedifferentiating (negative) markers of hyaline cartilage (Collagen I and Collagen X) in order to ascertain the extent of maturity and stability of the engineered cartilages. Collagen I was evaluated, but due to the unavailability of sheep Collagen X primer and antibody at the time of this study, we were unable to verify the hypertrophic status of these engineered cartilages. The last limitation to this study is the inability to circumscribe the injected cells to the lesion site only, to ensure better regeneration. This, if accomplished in the future, will help to save the precious cells only to the desired location.

## Conclusions

In this study ADSCs had better cell proliferations, but BMSCs had better chondrogenic inductions and gene expressions. There was no difference of ICRS score between ADSCs and BMSCs treatment. The presence of the tracking dye PKH26 at the resected portions of the neo-cartilages confirmed the participation of our injected cell in the osteoarthritic regeneration, hence both autologous chondrogenically induced ADSCs and BMSCs could be promising cell sources for cartilage regeneration in osteoarthritis.
